# Adolescent maturation of inhibitory inputs onto cingulate cortex neurons is cell-type specific and TrkB dependent

**DOI:** 10.3389/fncir.2015.00005

**Published:** 2015-02-16

**Authors:** Angela Vandenberg, David J. Piekarski, Natalia Caporale, Francisco Javier Munoz-Cuevas, Linda Wilbrecht

**Affiliations:** ^1^Neuroscience Graduate Program, University of CaliforniaSan Francisco, CA, USA; ^2^Department of Psychology, University of CaliforniaBerkeley, CA, USA; ^3^Department of Pharmacology, University of MarylandBaltimore, MD, USA; ^4^Helen Wills Neuroscience Institute, University of CaliforniaBerkeley, CA, USA

**Keywords:** cell type specificity, GABA, miniature post-synaptic currents, mouse, prefrontal cortex

## Abstract

The maturation of inhibitory circuits during adolescence may be tied to the onset of mental health disorders such as schizophrenia. Neurotrophin signaling likely plays a critical role in supporting inhibitory circuit development and is also implicated in psychiatric disease. Within the neocortex, subcircuits may mature at different times and show differential sensitivity to neurotrophin signaling. We measured miniature inhibitory and excitatory postsynaptic currents (mIPSCs and mEPSCs) in Layer 5 cell-types in the mouse anterior cingulate (Cg) across the periadolescent period. We differentiated cell-types mainly by Thy1 YFP transgene expression and also retrobead injection labeling in the contralateral Cg and ipsilateral pons. We found that YFP− neurons and commissural projecting neurons had lower frequency of mIPSCs than neighboring YFP+ neurons or pons projecting neurons in juvenile mice (P21–25). YFP− neurons and to a lesser extent commissural projecting neurons also showed a significant increase in mIPSC amplitude during the periadolescent period (P21–25 vs. P40–50), which was not seen in YFP+ neurons or pons projecting neurons. Systemic disruption of tyrosine kinase receptor B (TrkB) signaling during P23–50 in TrkB_F616A_ mice blocked developmental changes in mIPSC amplitude, without affecting miniature excitatory post synaptic currents (mEPSCs). Our data suggest that the maturation of inhibitory inputs onto Layer 5 pyramidal neurons is cell-type specific. These data may inform our understanding of adolescent brain development across species and aid in identifying candidate subcircuits that may show greater vulnerability in mental illness.

## Introduction

Inhibitory circuits in the prefrontal cortex are known to synchronize the firing of excitatory projection neurons (Cardin et al., 2009; Sohal et al., 2009) as well as influence synaptic plasticity (Sakata et al., 2009), both of which are thought to modulate executive functions such as behavioral regulation, flexibility, and working memory (Gonzalez-Burgos et al., 2011; Le Magueresse and Monyer, 2013). Developmental changes in the inhibitory circuitry regulate sensitive periods and may play a key role in the onset of psychiatric disorders (Lewis et al., 1999, 2005; Rubenstein and Merzenich, 2003; Hashimoto et al., 2004; Sohal et al., 2009). Brain-derived neurotrophic factor (BDNF) and its receptor TrkB are known to play a role in the maturation of inhibition during post-natal life (McAllister et al., 1999; Hensch, 2005) and deficits in these factors are linked to multiple late-onset psychiatric disorders, including schizophrenia and mood disorders (Rubenstein and Merzenich, 2003; Hashimoto et al., 2004; Lewis et al., 2005). While significant progress has been made in our understanding of the organization and development of inhibitory circuits in mammalian prefrontal cortex (Lewis et al., 2005; Otte et al., 2010; Fino and Yuste, 2011; Packer and Yuste, 2011; Krook-Magnuson et al., 2012; Le Magueresse and Monyer, 2013; Lee et al., 2014a,b), the precise connectivity of these inhibitory circuits remains controversial. In particular, there are still conflicting reports whether local GABAergic circuits differentially regulate specific excitatory subnetworks in the frontal cortex (Lewis et al., 2005; Otte et al., 2010; Fino and Yuste, 2011; Packer and Yuste, 2011; Krook-Magnuson et al., 2012; Le Magueresse and Monyer, 2013; Lee et al., 2014a,b). In this context, it is also unknown whether neurotrophin signaling acts to regulate the maturation of inhibition onto different neuron subtypes in a homogenous or specific fashion. Cell-type and circuit specific organization and vulnerability may be particularly important for understanding the etiology of psychiatric disease.

To investigate the differences in input onto different Layer 5 projection neurons of mouse anterior cingulate (Cg) cortex, we made whole-cell patch recordings of miniature excitatory (mEPSCs) and inhibitory (mIPSCs) postsynaptic currents in projection neurons identified using two strategies. First, we made use of the Thy1-YFPH mouse line that expresses YFP in a subset of Layer 5 pyramidal neurons under the regulation of the Thy1 promoter (Feng et al., 2000; Sugino et al., 2006). In the anterior Cg, YFP+ and YFP− neurons differ in their expression of Thy1 and numerous other genes (Sugino et al., 2006). A major proportion of YFP+ neurons send descending axons to the pons, spinal cord, and pyramidal tract (sometimes defined as PT-type neurons; Miller et al., 2008; Porrero et al., 2010), whereas a majority of YFP− Layer 5 neurons are thought to correspond to populations that project within the cortex and corpus callosum (Intratelencephalic, IT-type; Porrero et al., 2010). However, this correspondence is not perfect: some PT-type neurons (~33%) do not express YFP while a very small percentage of IT type neurons do (~2%) (Porrero et al., 2010). In a second strategy, we injected fluorescent retrobeads in the ipsilateral pons and the contralateral anterior Cg to label pons (Pons) and commissural (COM) projecting neurons, respectively, in the same brain slices.

We found that while mEPSC measures did not differ between YFP+ and YFP− neurons, YFP+ and Pons projecting neurons had a significantly higher mIPSC frequency compared to adjacent YFP− or COM projecting neurons. We also found that the YFP− population showed a TrkB dependent increase in mIPSC amplitude over periadolescent development, while YFP+ did not. Knowledge of this kind can help to isolate candidate subcircuits to better understand the etiology of psychiatric diseases that show onset during late development.

## Materials and methods

### Animals

**Figure 1:**
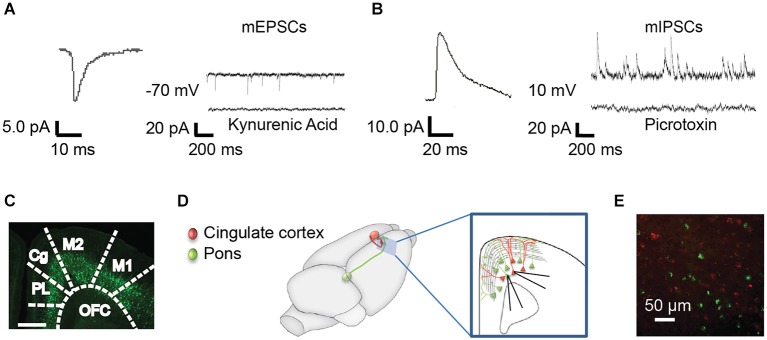
**Methods for recording mPSCs from identified subpopulations of Layer 5 neurons. (A)** Miniature excitatory post synaptic currents (mEPSCs) were recorded at −70 mV in the presence of TTX. Representative curve from 100 averaged events (left) and representative individual trace (right, top). mEPSCs were abolished with the nonspecific glutamate antagonist, kynurenic acid (right, bottom). **(B)** Miniature inhibitory post synaptic currents (mIPSCs) were recorded in the same neurons at 10 mV. Representative averaged event (left, *n* = 100) and trace (right, top). mIPSCs were abolished by the GABA_A_ receptor antagonist, picrotoxin (right, bottom). **(C)** mPSCs were recorded from YFP+ and YFP− pyramidal cells in the anterior cingulate (Cg) of Thy-1 YFPH line mice. PL, prelimbic; OFC, orbitofrontal cortex; M2, secondary motor cortex; M1, primary motor cortex (Franklin and Paxinos, 2008). **(D)** As an alternative method, colored retrograde beads were injected into contralateral Cg (red) or ipsilateral pons (green) in WT mice, allowing us to record from pons-projecting (Pons) and commissural (COM) Cg-projecting neurons within the same slice (inset). All recordings were targeted to Layer 5 of the Cg region in the right hemisphere. **(E)** Photo of red and green labeled pyramidal neurons in the Cg cortex.

All experimental procedures were approved by the Animal Care and Use Committees of the Ernest Gallo Clinic and Research Center and University of California at Berkeley. Wild-type (WT) (*n* = 30) and Thy1-eYFP line H mice (YFP−H) (*n* = 20) (Jackson Labs, line 003782) (Feng et al., 2000) were used at two ages (mixed sex). “Juvenile” group mice were P21–25, this is within 5 days of weaning, which is pre-puberty onset in mice, and “adolescent” mice were P40–50, typically post-puberty onset in mice. Sex differences in mPSC amplitude and frequency at each age were not found (data not shown). In the YFP−H line, only a subset of Layer 5 pyramidal neurons is labeled (Feng et al., 2000; Sugino et al., 2006; Figure 1C). YFP+ and YFP− Layer 5 neurons have previously been shown to have different firing properties (including differences in spike adaptation) (Sugino et al., 2006; Hattox and Nelson, 2007; Miller et al., 2008; Yu et al., 2008), and projections to different targets (Porrero et al., 2010).

In a second line of experiments, TrkB_F616A_ mice (Chen et al., 2005) were crossed to the Thy1 YFPH line. In the TrkB_F616A_ line, a phenylalanine to alanine substitution in the ATP binding pocket of the receptor allows for the temporally specific inhibition of TrkB when it is in the presence of the small molecule inhibitor 1NM-PP1 (Chen et al., 2005). Importantly, the mutation is functionally silent without the inhibitor (Chen et al., 2005; Kaneko et al., 2008). Mice that were homozygous TrkB_ F616A_ mutants with transgenic expression of YFP (*n* = 11) were used to study the impact of blocking TrkB signaling during a specific window of development using 1NM-PP1 (Chen et al., 2005).

### Injection of retrogradely transported microspheres

Injections were conducted under deep isoflurane anesthesia (1.5–3% in oxygen) in a stereotaxic apparatus. Red and/or green fluorescent retrobeads (undiluted, LumaFluor, Inc.) were injected into the left Cg cortex and/or right pons (PN) using a nanoliter injector (Nanoject, Drummond). Coordinates used were (in millimeters relative to bregma): Cg: 2.1 anterior-posterior (AP), 0.4 mediolateral (ML), and 0.5, 0.7 and 1.0 dorsoventral (DV); PN: −4.26 AP, 0.4 ML, and 4.6 DV (Franklin and Paxinos, 2008). Slice electrophysiology was performed at least 72 h after injection.

### 1NM-PP1 and minipump implantation

1NM-PP1 (Cayman Chemical, Ann Arbor MI) or vehicle solution (4% (vol/vol) DMSO and 2% (vol/vol) Tween-20 in saline) was administered systemically via osmotic minipumps (Alzet, Cupertino CA) implanted subcutaneously at the neck at P23. Pumps remained in place until sacrifice for electrophysiology (before P51). 1NM-PP1 was delivered at the rate of 6.25 nmol/h (Kaneko et al., 2008).

### Slice preparation

Mice were deeply anesthetized with a lethal dose of ketamine and xylazine and transcardially perfused with ice-cold cutting solution containing (in mM): 110 choline-Cl, 2.5 KCl, 7 MgCl_2_, 0.5 CaCl_2_, 25 NaHCO_3_, 11.6 Na-ascorbate, 3 Na-pyruvate, 1.25 NaH_2_PO_4_, and 25 D-glucose, and bubbled in 95% O_2_/ 5% CO_2_. Coronal sections (300 μm thick) were cut in ice-cold cutting solution and then transferred to ACSF containing (in mM): 120 NaCl, 2.5 KCl, 1.3 MgCl_2_, 2.5 CaCl_2_, 26.2 NaHCO_3_, 1 NaH_2_PO_4_ and 11 Glucose. Slices were bubbled with 95% O_2_/ 5% CO_2_ in a 35°C bath for 30 min, and allowed to recover for at least 30 min at room temperature before recording.

### Electrophysiology

**Figure 2:**
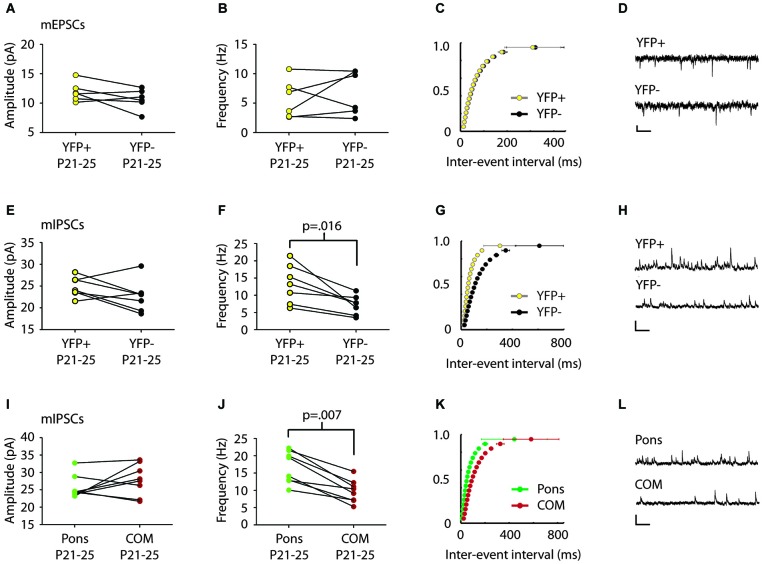
**mIPSC frequency differs in comparison of pairs of Layer 5 pyramidal cell subtypes. (A,B)** Comparison of mEPSCs between serially recorded pairs (*n* = 7) of YFP+ (yellow) and YFP− (black) neurons revealed that mEPSC amplitude **(A)** and frequency **(B)** were similar across pyramidal cell types (*p* > 0.5 Wilcoxon). **(C)** Cumulative frequency distribution plot of mEPSC inter-event interval (ISI) in serially recorded pairs of YFP+ and YFP− neurons. No significant difference was found between cell types (*p* > 0.05 KS test). **(D)** Sample traces. Scale: 200 ms, 10 pA.** (E)** Comparison of mIPSCs between serially recorded pairs (*n* = 7) of YFP+ (yellow) and YFP− neurons (black) revealed that mIPSC amplitudes were higher in YFP+ neurons but the difference was not significant beyond the trend level (*p* = 0.10 Wilcoxon). **(F)** mIPSC frequency was higher in YFP+ neurons than in YFP− neurons (*p* = 0.016 Wilcoxon). **(G)** A cumulative plot of ISI shows significant difference in mIPSC frequency between neuron types (*p* < 0.0001 KS-test). **(H)** Sample traces. Scale: 200 ms, 40 pA.** (I)** Pairs (*n* = 8) of adjacent cortico-pontine (Pons, green) and commissural (COM, red) projecting neurons, had similar mIPSC amplitude (*p* = 0.38 Wilcoxon), but different mIPSC frequency **(J)**, with more frequent mIPSCs in Pons neurons (*p* = 0.007 Wilcoxon). **(K)** A cumulative plot of ISI shows a significant difference in mIPSC frequency between neuron types (*p* < 0.0001 KS-test). **(L)** Sample traces. Scale: 200 ms, 40 pA.

**Figure 3:**
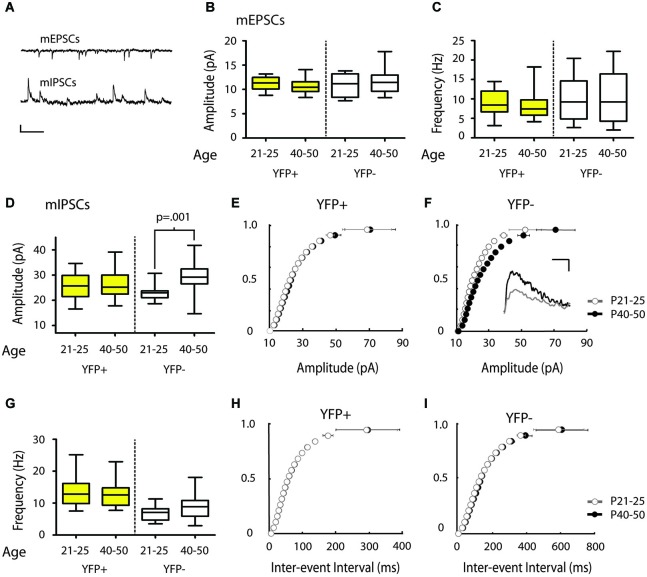
**mIPSC amplitude increases over the periadolescent period (P21–25 to P40–50) in YFP− neurons in the mouse Cg. (A)** Sample traces of mEPSCs (top) and mIPSCs (bottom) recorded from the same neuron. Scale top: 500 ms, 10 pA. Scale bottom: 500 ms, 20 pA.** (B,C)** There were no significant changes in mEPSC amplitude **(B)** or frequency **(C)** in the YFP+ or YFP− neuronal subtypes over the periadolescent period (amplitude: YFP+ P21–25 *n* = 17, animals = 5, P40–50 *n* = 28, animals = 14, *p* = 0.09; YFP− P21–25 *n* = 8, animals = 3, P40–50 *n* = 17, animals = 10, *p* = 0.98; frequency: YFP+ *p* = 0.22; YFP− *p* = 0.89, all *p* values from Mann Whitney). **(D)** Layer 5 YFP− neurons showed a 28% increase in mean mIPSC amplitude over the periadolescent period (P21–25 *n* = 10, animals = 3, P40–50 *n* = 19, animals = 10; *p* = 0.001 Mann Whitney). No significant age-related increase was found in YFP+ neurons (P21–25 *n* = 22, animals = 6, P40–50 *n* = 24, animals = 13, *p* = 0.58 Mann Whitney). **(E)** Cumulative probability plots of mIPSC amplitude in YFP+ populations showed a small, but significant, increase in the amplitude distribution from P21–25 to P40–50 (*p* = 0.03 KS test). **(F)** Cumulative probability plots of mIPSC amplitude in YFP− neurons show a strong rightward shift with development from P21–25 to P40–50 (*p* < 0.0001 KS test). Inset: Sample traces of IPSCs. Scale: 5 ms, 10 pA. **(G)** Average values of mIPSC frequency in YFP+ neurons showed no change with age (*p* = 0.75 Mann Whitney), while YFP− neurons showed a trend toward increasing mIPSC frequency (*p* = 0.09 Mann Whitney). **(H)** Cumulative probability plots of ISI showed no increase in mIPSC frequency over the periadolescent period in YFP+ populations, and a significant increase in mIPSC frequency for YFP− populations **(I)** over the same period (*p* < 0.0001 KS test). Box and whisker plots shown in **(B–D,G)**: Box indicates median (center bar) and first and third quartiles. Whiskers indicate data minimum and maximum.

**Figure 4:**
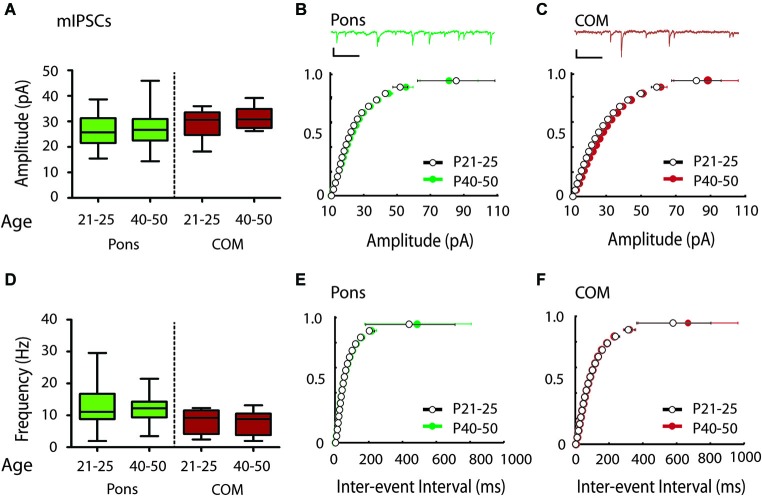
**Modest shift in the distribution of mIPSC amplitudes over the periadolescent period (P21–25 to P40–50) in COM projecting neurons in the mouse Cg. (A)** Layer 5 Pons projecting neurons showed no significant increase in mean mIPSC amplitude over the periadolescent period (P21–25 *n* = 15, animals = 4, P40–50 *n* = 16, animals = 6, *p* = 0.74 Mann Whitney). A small increase in the mean mIPSC amplitude of COM projecting neurons was not statistically significant (P21–25 *n* = 15, animals = 4, P40–50 *n* = 14, animals = 5, *p* = 0.40 Mann Whitney). **(B)** Top: Sample trace. Scale: 500 ms, 20 pA. Bottom: Cumulative probability plots of mIPSC amplitude in Pons projecting neurons showed no change with age. **(C)** Top: Sample trace. Scale: 500 ms, 20 pA. Bottom: Cumulative probability plot of mIPSC amplitudes in COM projecting neurons revealed a significant increase in distribution of mIPSC amplitude in COM neurons from P21–25 to P40–50 (*p* = 0.0003 KS test). **(D)** Plots of mIPSC frequency. Layer 5 Pons projecting neurons showed no significant changes in mean mIPSC frequency with age (P21–25 *n* = 15, animals = 4, P40–50 *n* = 16, animals = 6, *p* = 0.94 Mann Whitney). There were also no significant changes in mean mIPSC frequency in COM projecting neurons (P21–25 *n* = 15, animals = 4, P40–50 *n* = 14, animals = 5, *p* = 0.97 Mann Whitney). **(E)** Cumulative probability plot of mIPSC ISI in Pons projecting neurons **(F)** Cumulative probability plot of mIPSC frequency in COM projecting neurons. **(E,F)** KS tests showed no significant change in mIPSC frequency from P21–25 to P40–50 (*p* > 0.05). Box and whisker plots shown **(A,D)**: Box indicates median (center bar) and first and third quartiles. Whiskers indicate data minimum and maximum.

**Figure 5:**
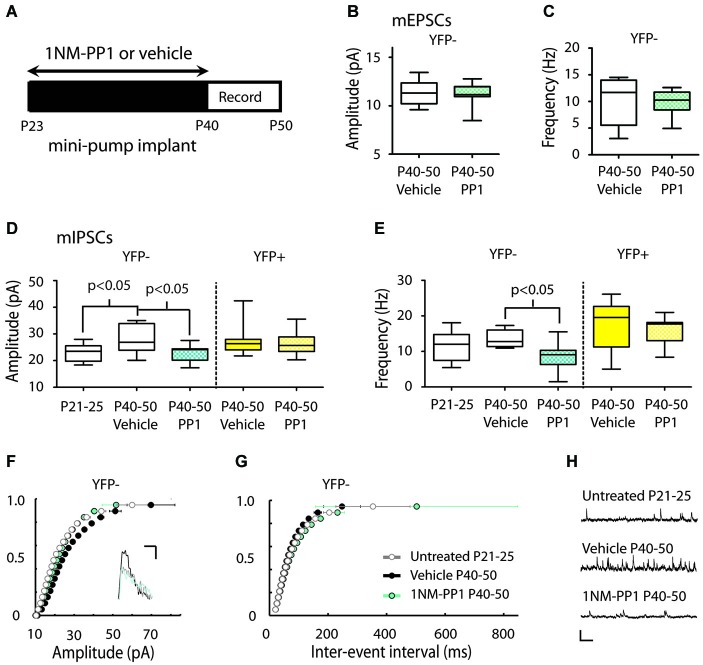
**Inhibiting TrkB blocks the periadolescent maturation of inhibition in YFP− neurons. (A)** Experimental timeline for periadolescent delivery of drug/vehicle by osmotic minipump implant. Measures of mEPSCs amplitude **(B)** and frequency **(C)** from YFP− neurons did not differ between vehicle (white) and 1NM-PP1 (blue) treated TrkB_F616A_xYFPH mice (amplitude: 1NM-PP1 *n* = 12, animals = 5; Vehicle, *n* = 6, animals = 3; *p* = 0.96; frequency: *p* = 0.54 Mann Whitney). **(D)** 1NM-PP1 treatment impacted mIPSC amplitude in TrkB_F616A_xYFPH mice (one-way ANOVA, *F*_(4,43)_ = 3.57, *p* = 0.01). In YFP− neurons, mean mIPSC amplitude in adolescent vehicle treated TrkB_F616A_xYFPH mice (*n* = 8, animals = 4) was 22% higher than in untreated juvenile TrkB_F616A_xYFPHmice (*n* = 9, animals = 2), replicating the developmental increase observed in Figure 3. The mean mIPSC amplitude in adolescent 1NM-PP1 treated TrkB_F616A_xYFPH mice (*n* = 13, animals = 4) was comparable to untreated juveniles and significantly lower (20%) than age-matched vehicle treated adolescent mice. In YFP+ neurons (yellow), mIPSC amplitude and frequency in 1NM-PP1 treated mice (*n* = 11, animals = 4) did not differ from age matched vehicle treated mice (*n* = 11, animals = 3). All *p*-values in the panel are Bonferroni *post hoc* comparisons. **(E)** 1NM-PP1 treatment impacted mIPSC frequency in TrkB_F616A_xYFPH mice (one-way ANOVA, *F*_(4,43)_ = 5.87, *p* < 0.001). *Post hoc* comparisons of mIPSC frequency between adolescent vehicle treated and periadolescent 1NM-PP1 treated (blue) TrkB_F616A_ xYFPH mice showed significant differences in YFP− neurons but not YFP+ neurons. **(F)** Cumulative probability plot of mIPSC amplitudes: the distribution from untreated juvenile TrkB_F616A_xYFPH mice (P21–25, gray and white) was significantly different from P40–P50 vehicle controls (black) (*p* < 0.0001 KS test, uncorrected for multiple comparisons). The distribution of amplitudes from 1NM-PP1 treated TrkBF_F616A_xYFPH mice (blue) overlapped with juveniles (*p* > 0.05) and showed a significant difference from age matched vehicle-treated TrkB_F616A_xYFPH controls (*p* < 0.0001 KS test, uncorrected). Inset: Sample traces. Scale: 5 ms, 10 pA. **(G)** Cumulative probability plots of ISIs in TrkB_F616A_ xYFPH mice: untreated juvenile TrkB_F616A_ mice (P21–25, gray and white) were significantly different from P40–P50 vehicle treated controls (black) (*p* < 0.0001 KS test, uncorrected). 1NM-PP1 treated TrkB_F616A_ xYFPH mice (blue) also showed a significant difference from age-matched vehicle treated TrkB_F616A_xYFPH controls (*p* < 0.0001 KS test, uncorrected). **(H)** Sample traces. Scale: 200 ms, 40 pA. Box and whisker plots shown **(B–E)**: Box indicates median (center bar) and first and third quartiles. Whiskers indicate data minimum and maximum.

Recordings were obtained from Layer 5 pyramidal neurons in the right hemisphere Cg cortex (Franklin and Paxinos, 2008). In most experiments (Figures 2, 3, 5) whole cell voltage clamp miniature excitatory post synaptic currents (mEPSCs; Figure 1A) were recorded at −70 mV and inhibitory post synaptic currents (mIPSCs) were recorded in the same neurons at 10 mV (Figure 1B). The bath solution contained 1 μM tetrodotoxin to block action potentials. Kynurenic acid and picrotoxin confirmed the currents recorded at these voltages were consistent with mEPSCs and mIPSCs, respectively (Figures 1A,B). Recording pipettes had a resistance of 3–4 MΩ and were filled with intracellular solution (in mM): 115 Cs-methanesulfonate, 10 HEPES, 10 BAPTA, 10 Na2-phosphocreatine, 5 NaCl, 2 MgCl2, 4 Na-ATP, 0.3 Na-GTP. In some experiments 30 μM of Alexa-594 was added to the internal solution in order to visualize recorded neurons. For a subset of experiments (Figure 4), mIPSCS were specifically isolated; cells were held at −70 mV with intracellular solution (in mM): 115 CsCl, 10 HEPES, 10 Na2-phosphocreatine, 5 NaCl, 2 MgCl2, 4 Na-ATP, 0.3 Na-GTP, 0.02 EGTA and 15 KCl and the bath solution contained 0.5 μM tetrodotoxin, 5 μM NBQX and 25 μm APV to block action potentials, AMPA/Kainate, and NMDA currents, respectively. All recordings were made using Multiclamp 700 B amplifier and were not corrected for liquid junction potential. Data were digitized at 10 kHz and filtered at 1 or 3 kHz using a Digidata 1440 A system with PClamp 10.2 software (Molecular Devices, Sunnyvale, CA, USA). Only cells with access resistance of <25 MΩ were included in the analysis. Access resistance was not corrected and was recorded every 30 s. Cells were discarded if parameters changed more than 20%. Data were analyzed using MiniAnalysis software (Synaptosoft, INC, GA). All experiments were conducted at 32°C. All chemicals were from Sigma or Tocris Biosciences with the exception of MgCl2 (Fluka) and 1NM-PP1 (Cayman).

### Statistics

Mann Whitney, Wilcoxon matched-pairs signed rank test, or one way analysis of variance (ANOVA) with Bonferroni *post hoc* tests were used to analyze statistical significance (Graphpad Prism). A two-sample Kolmogorov-Smirnov (KS) test (Matlab, Mathworks) was used to compare cumulative distributions. Data displayed in cumulative distributions were discretized into twenty equally sized bins that span the entire distribution of recorded values. Differences were considered significant if *p* < 0.05. All results are reported as the mean ± standard error of the mean (SEM) unless otherwise noted.

## Results

### Cell type specific differences in mIPSCs in Layer 5 of the cingulate cortex

We first examined whether synaptic inputs differ between different types of projection neurons in Layer 5 of the Cg cortex. We compared mPSC amplitude and frequency in serially recorded pairs of YFP+ and YFP− neurons in juvenile mice (P21–25). For two neurons to be considered a pair their cell bodies had to be within 200 microns of one another. We found no significant difference in mEPSCs between cell types (Amplitude: YFP+ 11.9 ± 0.7 pA, YFP− 10.7 ± 0.7 pA; Frequency: YFP+ 5.8 ± 1.3 Hz, YFP− 6.8 ± 1.5 Hz; *p* > 0.5 Wilcoxon) (Figures 2A–C). However, there was a significant difference in mIPSC frequency between adjacent YFP+ and YFP− cells (YFP+ 13.3 ± 2.1 Hz, YFP− 7.2 ± 1.1 Hz; *p* = 0.016 Wilcoxon) (Figures 2F,G).

We next used green and red retrobeads (Figures 1D,E) to identify neurons that project to the pons nucleus (abbreviated as Pons) and neurons that project to the Cg of the opposite hemisphere (commissural cells, abbreviated as COM). Recording from serial pairs of adjacent Pons and COM neurons in P21–25 WT mice, we found that again, while there was no difference in mIPSC amplitude between cell types (Pons 25.7 ± 1.9 pA vs. COM 27.9 ± 1.6 pA; *p* = 0.38 Wilcoxon) (Figure 2I), mIPSC frequency was significantly lower in COM neurons compared to Pons (Pons 16.7 ± 1.6 Hz vs. COM 9.8 ± 1.2 Hz; *p* = 0.007 Wilcoxon) (Figures 2J,K).

### mIPSC amplitude increases in YFP− but not YFP+ neurons during adolescence

We next determined whether inputs onto different subtypes of Layer 5 Cg pyramidal neurons change during adolescent development. To this end, we compared mPSC recordings made in juvenile mice (P21–25) with recordings made in adolescent mice (P40–50). Comparisons of mEPSC measures revealed no significant changes from P21–25 to P40–50 in YFP+ (Amplitude: 11.26 ± 0.32 vs. 10.63 ± 0.25 pA, Frequency: 9.00 ± 0.84 vs. 8.12 ± 0.62 Hz, younger vs. older) or YFP− neurons (Amplitude: 11.05 ± 0.85 vs. 11.63 ± 0.59 pA, Frequency 10.05 ± 2.09 vs.10.58 ± 1.60 Hz) (Figures 3B,C). However, in YFP− neurons, from P21–25 to P40–50, there was a statistically significant 28% increase in mIPSC amplitude (22.97 ± 1.04 vs. 29.46 ± 1.31 pA, *p* < 0.001 Mann Whitney) (Figures 3D,F). A significant increase in mIPSC frequencies over this time was also apparent in the cumulative probability plot (*p* < 0.01 KS test) (Figure 3I), but only reached trend level when comparing mean mIPSC frequency (6.90 ± 0.76 vs. 9.78 ± 1.03 Hz, *p* = 0.09; Mann Whitney) (Figure 3G). In the population of YFP+ neurons, mIPSC amplitude (Figures 3D,E) and frequency (Figures 3G,H) did not show changes over this developmental period (Amplitude 25.70 ± 1.10 vs. 26.73 ± 1.16 pA; Frequency 13.52 ± 0.97 vs. 12.96 ± 0.81 Hz).

To determine if Pons and commissural projecting COM neurons show a similar developmental profile to YFP+ and YFP− neurons, we also recorded mIPSCs from Pons and COM neurons in juvenile and adolescent WT mice (Figure 1). Consistent with YFP+ neurons, in Pons projecting neurons we found that there was no developmental change in mIPSC amplitude or frequency from P21–25 to P40–50 (Amplitude 26.28 ± 1.78 vs. 27.13 ± 1.75 pA, Figures 4A,B; Frequency:12.70 ± 1.80 vs.12.53 ± 1.23 Hz, Figures 4D,E). Consistent with YFP− neurons, in COM projecting neurons we did observe a significant rightward shift in the distribution of mIPSC amplitudes with age (*p* < 0.001) (Figure 4C). However, this difference was not apparent when comparing the group mean mIPSC amplitude (Figure 4A) (29.05 ± 1.39 vs. 31.40 ± 1.13 pA, *P* = 0.40 two tailed Mann Whitney, an 8% increase). COM neurons did not show changes in mIPSC frequency from P21–25 to P40–50 (7.97 ± 0.92 vs. 7.93 ± 1.00 Hz, younger vs. older) (Figures 4D,F).

### Periadolescent disruption of TrkB signaling stalls maturation of mIPSCs in YFP− neurons

We next studied the putative role of TrkB signaling in the developmental increase of mIPSCs in Layer 5 neurons using a chemical-genetic approach to inhibit TrkB signaling with the small molecule inhibitor 1NM-PP1 in TrkB_F616A_xYFPH mice (Chen et al., 2005) (see Section Materials and Methods). We used osmotic minipumps to systemically deliver either 1NM-PP1 or vehicle (DMSO in saline) in these mice from P23 to the day of recording (between P40–50) (Figure 5A).

There was no effect of 1NM-PP1 treatment on mEPSC measures in P40–50 YFP− neurons (vehicle amplitude 11.34 ± 0.54 pA, frequency 10.18 ± 1.89 Hz; 1NM-PP1 amplitude 11.19 ± 0.31 pA, frequency 9.93 ± 0.64 Hz) (Figures 5B,C). We also replicated mIPSC results first observed in YFPH line (Figure 3) in the TrkB_F616A_xYFPH mice. We found vehicle treated TrkB_F616A_xYFPHmice showed the expected developmental increase in mIPSC amplitude in YFP− Layer 5 neurons (P21–25 untreated: 23.03 ± 1.09 vs. P40–50 vehicle: 28.04 ± 1.89 pA, 22% increase) (Figures 5D,F). When TrkB_F616A_xYFPH mice were treated with 1NM-PP1 from P23 to P40–50, the developmental increase in mIPSC amplitude was not observed (P40–50 1NM-PP1 22.86 ± 0.91 pA) (Figures 5D,F). A Bonferroni *post hoc* test revealed that mIPSC amplitudes in YFP− neurons in P40–50 1NM-PP1 treated mice were significantly smaller than those found in age and genotype matched vehicle treated controls (*p* < 0.05). Furthermore, the mIPSC amplitudes observed were comparable to those of untreated juvenile TrkB_F616A_xYFPH mice (*p* > 0.05).

Comparisons between groups also revealed treatment related differences in mIPSC frequency in TrkB_F616A_xYFPH mice (P21–25 untreated: 11.40 ± 1.43, P40–50 vehicle: 13.46 ± 0.85, P40–50: 1NM-PP1 8.62 ± 1.09 Hz) (Figures 5E,G). A Bonferroni *post hoc* test showed that mIPSC frequency in YFP− neurons in adolescent 1NM-PP1 treated mice was lower than in the adolescent vehicle treated control group (*p* < 0.05, Figures 5E,G).

In YFP+ neurons in P40–50 TrkB_F616A_xYFPH mice, mIPSC amplitude (vehicle 27.31 ± 1.57 pA, 1NM-PP1 26.84 ± 1.32 pA) (Figure 5D) and frequency (vehicle 17.88 ± 1.90 Hz, 1NM-PP1 16.25 ± 1.15 Hz) (Figure 5E) were not affected by 1NM-PP1 treatment (Bonferroni *post hoc p* > 0.05).

To ensure that 1NM-PP1 did not have unanticipated off-target effects we also recorded mIPSCs in YFP− neurons in WT mice treated with either 1NM-PP1 or vehicle over the same periadolescent period as TrkB_F616A_xYFPH mice. At P40–50, we found no significant difference in mIPSC amplitude between vehicle and 1NM-PP1 treated WT mice (vehicle 27.31 ± 1.57 pA, *n* = 12; 1NM-PP1 26.08 ± 1.89 pA, *n* = 8; *p* = 0.31 Mann-Whitney) (data not shown).

## Discussion

Reorganization of the prefrontal circuits during the periadolescent period may underlie vulnerability to psychiatric disorders such as schizophrenia and mood disorders (Spear, 2000; Chambers et al., 2003; Gonzalez-Burgos et al., 2008), both of which show onset typically during the periadolescent period (Paus et al., 2008). Current theories suggest that these disorders may be the result of imbalances in excitation and inhibition (Rubenstein and Merzenich, 2003; Lewis et al., 2005; Gonzalez-Burgos et al., 2008; Yizhar et al., 2011) and/or deficits in long range cortical-cortical communication, particularly in the case of schizophrenia (Friston, 1998; Andreasen, 1999; Uhlhaas and Singer, 2010, 2011; Whitford et al., 2010).

We measured mPSCs onto Layer 5 pyramidal neurons that express different genes and project to different targets (Figure 1). We found that YFP+ neurons in the Thy1 YFPH line have higher mIPSC frequency rates than adjacent YFP− neurons, and similarly Pons projecting neurons show higher frequencies than COM projecting neurons (Figure 2). In addition, our results show that during the adolescent transition to young adulthood, there is a shift toward larger amplitude inhibitory currents on YFP− and to smaller extent COM-projecting pyramidal neurons (Figures 3F, 4C, 5F). The maturation of inhibitory synapses on YFP− neurons can be blocked by disruption of TrkB signaling without affecting mEPSCs in the same neurons or inhibitory synapses on neighboring YFP+ neurons (Figure 5).

Previous studies have establish the importance of BDNF/TrkB in the development of inhibition, as well as triggering the normal opening and closing of the critical period for ocular dominance plasticity (Abidin et al., 2008; Kaneko et al., 2008; Gogolla et al., 2009). In hippocampal cultures BDNF has been shown to increase GABA_A_ receptor expression after 48 h of treatment (Yamada et al., 2002). Moreover, a mutation that disrupts the ability of CREB to bind to BDNF promoter IV results in deficits in miniature IPSCs, reduced expression of GABAergic markers, and fewer inhibitory synapses in cultured cortical neurons (Hong et al., 2008). Mice that lack promoter IV BDNF transcription show decreased PV staining in the PFC as well as deficits in GABAergic, but not glutamatergic, synaptic transmission (Sakata et al., 2009). TrkB hypomorphic mice also show a gene dependent decrease in both GAD67 and PV mRNA (Hashimoto et al., 2005). Moreover, in BDNF heterozygous KO mice the overall balance in the strength of cortical excitation to inhibition is shifted towards decreased inhibition (Abidin et al., 2008). These studies suggest that BDNF plays an important role in regulating the balance of inhibition and excitation (I/E) through activity-dependent positive or negative feedback loops. Our data suggests that the development of inhibition over peri-adolescence, and its dependence on TrkB signaling, may be cell-type specific rather than homogenous in nature.

Studies on local cortical circuit organization have emphasized the local homogeneity of inhibitory neuron connectivity across multiple regions in cortex (Packer and Yuste, 2011) and the stability of these patterns with age (Fino and Yuste, 2011; Sahara et al., 2012). Conclusions from these studies seem to conflict with others that find evidence for circuit specific differences in inhibitory connectivity (Yoshimura and Callaway, 2005; Yoshimura et al., 2005; Otte et al., 2010; Krook-Magnuson et al., 2012; Lee et al., 2014a,b). While the methods used here cannot resolve whether the presynaptic targeting of specific interneuron subtype axons is selective or indiscriminate, our data show that inhibitory synapses on the Layer 5 neurons of Cg cortex can differ even when cells are adjacent (Figure 2). Although, we cannot yet discriminate whether these differences are due to origin of the synapses, changes in the number of inhibitory synapses and/or the probability of release of those synapses, our data suggest that subcircuits of the Cg cortex are differentially regulated by inhibition depending on their gene expression and projection target. These data are consistent with findings in other cortical regions (Anderson et al., 2010; Varga et al., 2010; Krook-Magnuson et al., 2012).

Across the neocortex, Layer 5 pyramidal neurons have previously been subdivided into roughly two classes: those with mainly intratelencephalic connections (IT-type), those that project subcortically to the pons, pyramidal tract (PT), and other subcortical targets (PT-type) (Reiner et al., 2010; Krook-Magnuson et al., 2012). PT-type neurons have no spike adaptation and present a complex apical tuft morphology. IT-type neurons, which project within the cerebral cortex including the contralateral hemisphere, show spike adaptation and tend to have a simpler apical tuft morphology (Hattox and Nelson, 2007; Miller et al., 2008; Yu et al., 2008; Sohal et al., 2009; Gee et al., 2012). YFP− and YFP+ neurons in the Thy1 YFPH line, on average, are consistent with IT and PT-type neurons (Hattox and Nelson, 2007; Miller et al., 2008; Yu et al., 2008), but the overlap is not perfect (Porrero et al., 2010). A simple interpretation of our data from YFP− and COM neurons together, suggests that inhibition onto IT-type neurons is changing during the periadolescent period in the Cg. Our data from YFP+ and Pons neurons suggest PT-type neurons do not show parallel changes over the period. However, it should be noted that the classification of pyramidal cells as either YFP−/YFP+ or IT or PT-type neurons is broad and these may also contain additional subtypes (Fame et al., 2011; Otsuka and Kawaguchi, 2011) that may follow different patterns of maturation. In the present study, the magnitude of the mIPSC amplitude difference observed with age was greater when we used lack of Thy1 YFP expression to identify a Layer 5 cell-type (YFP− 28% increase) vs. retrobeads injected into the opposite hemisphere (COM 8% increase). It is possible these two identification strategies sampled from different subpopulations of Layer 5 cells. In our retrograde bead tracing strategy we specifically labeled Layer 5 pyramidal cells that projected either to the ipsilateral pons or the contralateral anterior Cg. In Thy 1 YFPH mice, YFP+ cells have been shown to project to the pons and to the thalamus and colliculus while YFP− cells may project to the contralateral Cg as well as other cortical regions. This may explain why age related changes in mIPSC amplitude observed in YFP− neurons were not matched in scale by changes in COM neurons and may argue for even greater cell-type specificity than we were able to resolve. It is also possible that expression of Thy1 itself and co-expression of other genes (see Sugino et al., 2006) may also impact cell to cell interaction and maturation state of the neurons (Tiveron et al., 1992; Barker and Hagood, 2009). Data such as ours may enhance interest in neural subtypes that do not express Thy1, a commonly used genetic marker. Future experiments will be necessary to resolve further sub-classification differences.

Despite some open questions about the total number of cell subtypes in Layer 5, the cell-type specificity revealed in our data may inform understanding of human brain development in both health and disease states. It has previously been proposed that alterations in inhibitory circuits potentially driven by deficits in BDNF/TrkB signaling could underlie the etiology of schizophrenia (Lewis et al., 1999, 2005, 2011; Chen et al., 2006; Gonzalez-Burgos et al., 2008; Gogolla et al., 2009). A study of postmortem human brains found that BDNF and TrkB expression in the prefrontal cortex area 9 were reduced in two cohorts of schizophrenics along with parvalbumin (PV) and the 67 kDa isoform of glutamate decarboxylase (GAD_67_; Hashimoto et al., 2005). Differences in TrkB and BDNF mRNA in schizophrenia patient/matched control pairs correlated positively with differences in GAD_67_ mRNA (Hashimoto et al., 2005). The correlation between TrkB and GAD_67_ mRNA was also confirmed in TrkB hypomorphic mice (Hashimoto et al., 2005). Studies in cultured neurons also show BDNF/TrkB signaling regulates inhibitory synapse formation (Rutherford et al., 1997; Baldelli et al., 2002). While the usual caveats associated with comparing cellular, animal and human studies apply, based on our results we hypothesize that disruption of the maturation of adolescent inhibition onto a subtype of cortical cell may result in dysfunction of the PFC and potentially contribute to the onset of mental illness.

Our data suggests that deficits in TrkB found in schizophrenics may possibly have greater impact on the maturation of IT-type neurons that connect cortical areas. This is consistent with data from schizophrenic patients that shows decreased synchrony between long-range cortical areas (Uhlhaas and Singer, 2010) and abnormalities of white matter in the cingulum bundle in particular (Kubicki et al., 2003; Whitford et al., 2010). Developmental studies in healthy human subjects also show changes in cortico-cortical functional connectivity, oscillatory activity and synchrony over the adolescent period (Fair et al., 2007; Uhlhaas and Singer, 2011). These studies together with our data suggest that there is inhibition-mediated remodeling of information processing between the frontal hemispheres across adolescence. We hypothesize that changes in inhibition on IT-type neurons could mediate changes in the synchronization between the two hemispheres, potentially contributing to changes in neural function related to schizophrenia and other late onset developmental disorders. Future studies investigating the etiology of mental illness associated with frontal cortex dysfunction may be aided by further knowledge of cell types within these regions.

## Author and contributors

Angela Vandenberg, David J. Piekarski and Francisco Javier Munoz-Cuevas collected and analyzed data, Natalia Caporale analyzed data, Linda Wilbrecht and Angela Vandenberg wrote the paper with input from all authors.

## Conflict of interest statement

The authors declare that the research was conducted in the absence of any commercial or financial relationships that could be construed as a potential conflict of interest.
